# Occurrence and enumeration of *Campylobacter spp*. during the processing of Chilean broilers

**DOI:** 10.1186/1471-2180-9-94

**Published:** 2009-05-15

**Authors:** Guillermo Figueroa, Miriam Troncoso, Cristián López, Patricia Rivas, Magaly Toro

**Affiliations:** 1 Laboratory of Microbiology, Institute of Nutrition and Food Technology, University of Chile, Macul 5540 Santiago, Chile

## Abstract

**Background:**

Thermotolerant *Campylobacter *is among the more prevalent bacterial pathogens that cause foodborne diseases. This study aimed at evaluating the occurrence of thermotolerant *Campylobacter *contamination in chicken carcasses and processing plant stations (chilling water, scalding water, defeathering machinery, evisceration machine, and transport crates) in two of the Chilean main slaughterhouses. In addition, the isolation rates of thermotolerant *Campylobacter *during evisceration and following chiller processing were compared.

**Results:**

The overall slaughterhouse contamination with thermotolerant *Campylobacter *was 54%. Differences were evident when the results from each plant were compared (plant A and plant B was 72% and 36%, respectively). The sampling points with the greatest contamination rates in both plants were after evisceration (90% and 54%, for plants A and B respectively). The decrease of thermotolerant *Campylobacter *contamination after chilling was significant (2 and 1.6 logs for plant A and B respectively P < 0.05).

**Conclusion:**

Our findings indicate that chilling process has a limited effect in the final products *Campylobacter *contamination because poultry enter the slaughter processing with high counts of contamination. This may represent a health risk to consumers, if proper cooking practices are not employed. The levels and frequencies of *Campylobacter *found during the processing of Chilean poultry appear to be similar to those reported elsewhere in the world.

## Background

Thermotolerant *Campylobacter *is a zoonotic bacteria and one of the main causes of gastroenteritis worldwide, including both developed and developing countries [1]. During 2006 *Campylobacter jejuni *was the second cause of sporadic gastroenteritis in the USA, with an incidence of 12.71 cases per 100.000 inhabitants [2]. It has also been reported that 80% of all *Campylobacter *related illnesses are transmitted through food and are responsible for no less than 5% of food-related deaths [3]. The two species commonly associated with enteric diseases are *Campylobacter jejuni *and *Campylobacter coli*, with *C. jejuni *being more frequent (80–90%) [1].

*Campylobacter *may be transferred to humans indirectly through the ingestion of contaminated water or food [4] and to a minor extent by direct contact with contaminated animals or animal carcasses. Despite the identification of numerous natural and artificial reservoirs for *Campylobacter *[5], most case-control studies seeking to identify the index source of infection, have identified poultry handling, processing, cooking, and/or preparation outside the home as significant contributing risk factors for disease [6,7]. *C. jejuni *infection typically results in an acute, self-limited gastrointestinal illness characterized by diarrhea, fever, and abdominal pain. The most significant post-infectious sequelae of *C. jejuni *infection is Guillain-Barre's syndrome (GBS). Occurrence data on *Campylobacter *positive chicken in Chilean processing plants is limited. The frequent presence of thermotolerant *Campylobacter*, and more specifically *C. jejuni *in broiler chickens, moved public health and international trade organizations to incorporate its control in the Hazard Analysis Critical Control Point (HACCP) system [8]. This strategy is aimed at identifying and controlling the presence of enteric pathogens in all stages of the food chain; particularly in the transport to and in the slaughterhouse processing [9,10]. FSIS recently proposed a new "risk-based inspection" approach supported by scientific risk assessment to provide the poultry industry with better options to control contamination in order to produce safe, unadulterated product [11]. To achieve these food safety objectives, more information of local origin about the epidemiology, physiology, and ecology of *Campylobacter *is urgently required.

This study was aimed to, a) identify thermotolerant *Campylobacter *contamination in broiler carcasses collected during poultry processing; b) identify thermotolerant *Campylobacter *contamination within poultry processing plants, c) compare the isolation rates of thermotolerant *Campylobacter *following the evisceration and chilling processes during commercial poultry preparation.

Our goals were to generate information to facilitate microbiological risk assessment studies necessary to reduce and control contamination by *Campylobacter *within the Chilean poultry industry and the development of interventional strategies in the approved HACCP plans.

## Results

Of the 625 samples analyzed (whole chicken, processing plant environment and caecal samples), thermotolerant *Campylobacter *were cultured in 338 (54%). This includes both poultry processing plants (plants A and B). The overall occurrence of thermotolerant *Campylobacter *contamination was significantly higher (P < 0.05) in plant A (72%) than in plant B (36%).

### Thermotolerant *Campylobacter *in chicken carcasses during processing

The data obtained from both plants are shown in Table 1. The whole chicken contamination rate with thermotolerant *Campylobacter *at plant A was 80%. This rate was significantly lower in the plant B (41%). The greatest contamination rate in both plants was after evisceration (90% and 54%, for plants A and B respectively) (Table 1).

**Table 1:** Occurrence of thermotolerant *Campylobacter *on chicken's broiler carcasses evaluated in 4 processing's stages in two Chilean slaughterhouses.

Plant	Reception	After defeathering	After evisceration	After chilling	Total
**A**	35/44 (80)	46/62 (74)^a^	61/68 (90)^b^	46/62 (68)^c^	188/236 (80)
**B**	22/48 (46)^a^	15/62 (24)^b^	37/68 (54)^c^	23/61 (38)	97/239 (41)

n° of sample positive/n° examined (%).

Within each row, letters indicates statistically significantly different (P < 0.05, Test of proportion)

The overall contamination rate (plants A and B) with thermotolerant *Campylobacter *in the chicken carcasses following evisceration was 72%; this rate decreased significantly (P < 0.05) after the carcasses were chilled in the water tanks (56%). The detection of thermotolerant *Campylobacter *after evisceration was 90% in plant A. This rate decreased significantly after chilling (68%) (P < 0.05, Chi-square test). In contrast, there was no decrease in plant B.

In an attempt to ascertain the pre-processing baseline thermotolerant *Campylobacter *microbial status, the caecal content of 40 chickens were analyzed. This analysis identified *Campylobacter jejuni *in 85% (17/20) and 25% (5/20) in plants A and B, respectively.

### Occurrence of thermotolerant *Campylobacter *contamination in processing plant environment samples

As shown in Table 2, the rates of thermotolerant *Campylobacter *present in chicken in each processing environment sample analyzed as a whole varied between 35% and 22% for plants A and B respectively. Otherwise, in both plants, the highest isolation rate of thermotolerant *Campylobacter *was found in the evisceration machine. This coincides with the greatest contamination rates observed after evisceration, as described earlier. Thermotolerant *Campylobacter *was isolated in only one sample of chilling water from a total of 22 samples analyzed (plant B).

**Table 2:** Occurrence of thermotolerant *Campylobacter *isolated from environment samples in two Chilean poultry slaughterhouses.

Plant	Defeathering machine	Evisceration machine	Scalding water	Chilling water	Transport crates	Total
**A**	4/11 (36)	7/11 (64)	2/11 (18)	0/11 (0)	6/11 (55)	19/55 (35)
**B**	3/11 (27)	4/11 (36)	1/11 (9)	1/11 (9)	3/11 (27)	12/55 (22)

n° of sample positive/n° examined (%)

### Enumeration of thermotolerant *Campylobacter*

To perform the bacterial counts only the positive samples were taken into account. The thermotolerant *Campylobacter *contamination found in carcasses collected after evisceration and after chilling is shown in Table 3. Overall, *C. jejuni *contamination, ranged from 3.3 log_10 _up to 7.7 log_10 _*cfu*/carcass. As expected, the plant that had carcasses with the highest numbers after evisceration also had carcasses with the highest numbers after chilling. The decreased of thermotolerant *Campylobacter *contamination following the chilling process was significant, 2 and 1.6 log_10 _for plants A and B respectively (P < 0.05, Kruskal-Wallis test). Despite this, samples collected after chilling with counts as high as 6.4 log_10 _*cfu*/carcass were observed in both plants.

**Table 3:** Counts of thermotolerant *Campylobacter *(with standard deviations) on chicken's carcasses sampled after evisceration and after chilling in two Chilean poultry slaughterhouses.

	Median (log *cfu*/carcass)			
	
Plant	n	After evisceration	n	After chilling
**A**	68	5.2 ± 1.1^a^	62	3.3 ± 0.9^b^
**B**	68	6.1 ± 1.2^a^	61	4.5 ± 0.9^b^

Within each row, letters indicates statistically significantly different (P < 0,05 Kruskal-Wallis test)

### Thermotolerant *Campylobacter *species and biotypes

Table 4 shows the biotypes of thermotolerant *Campylobacter *recovered from plants A and B for all the sampling points tested. *C. jejuni *was the species most frequently isolated (627/645, 97%), whereas *C. coli *accounted for 18/645 (3%) of the strains collected. *C. jejuni *biotyping tests showed that biotype II was by far most prevalent in both plants (573/645, 89%). The remaining strains belonged to biotypes IV (30/645, 5%), and I (24/645, 4%). Biotype *C. jejuni *II was most frequently isolated from carcasses, processing plant environment, and caecal contents during processing. Additionally, only a few strains were *C. coli *biotypes II (2%) and I (1%).

**Table 4:** Sources and distribution of *Campylobacter *biotypes isolated from chickens carcasses, environmental samples and caecal contents in two Chilean poultry slaughterhouses.

	Plant A				Plant B			
	
Strains	Chicken carcasses	Environment	Caecal contents	Total	Chicken carcasses	Environment	Caecal contents	Total
	
C. coli biotype I	6	1	1	8				
C. coli biotype II	8			8	2			4
C. jejuni biotype I	14			14	9	1		10
C. jejuni biotype II	305	26	25	356	187	22	8	217
C. jejuni biotype IV	18	2	4	24	4	2		6
**Total**				**410**				**235**

## Discussion

In this study, as showed in table 1 and 2 thermotolerant *Campylobacter *contamination is widespread in caecal contents, processing plant environment and the poultry carcasses that reach the retailers stores. In pioneering initial studies conducted in 1982, Figueroa et al. [12], found that the *C. jejuni *bacterial load in the cloacal contents of 51 chickens (21 processed and 26 live birds) was fairly high: 46 specimens (90%); 25 (96%) in live birds and 21 (84%) in processed birds. Recent studies (Figueroa A., unpublished results) revealed much lower prevalence rates (12%) in some processed birds analyzed with a similar methodology, suggesting that carcasses decontamination can be reached. Despite this *C. jejuni *is sought as the most frequent pathogen isolated from poultry meats in Chile [13].

Microbiological analysis during poultry processing in slaughterhouses confirmed previous reports by Stern et al. [14] and Arsenault et al. [15] who observed a positive correlation between the contamination of carcasses and the high positivity rates for *Campylobacter *of flocks at the farm level. The recovery rates of *Campylobacter *in plant B represent lower contamination rates in both cloacal swabs and caecal content samples at plant A. This disparity in the intestinal tract colonization in live birds may explain the differences in the positive rates found in poultry carcasses and the environment samples between both plants resulting in an increased cross contamination risk during slaughter and processing.

The proportion of carcasses contaminated with *Campylobacter *increase during evisceration steps. This findings was corroborated by the fact that the number of positive carcasses increased significantly (P < 0.05) after evisceration. Rosenquist et al. [16] observed that as an average the evisceration process led to a significant increase in the numbers of *Campylobacter *by 0.5 log_10 _CFU/g of neck skin. The increase in contaminated carcasses is a result of viscera rupture, inevitably leading to the contamination of equipment, working surfaces, process water, and air and increasing the opportunities for cross contamination of *Campylobacter*-free carcasses during processing [5]. As the machinery used cannot adapt to the natural variation in the size of the carcasses being processed, the rupture of the intestines and the leak of fecal material is not uncommon in the slaughter plants [16,17]. Based on the results presented here, we may conclude as previously reported, that evisceration is a critical step in carcass contamination [5,16,18].

The immersion chilling procedure has been identified as a critical control point (CCP) in a generic Hazard Analysis Critical Control Points (HACCP) study of poultry contamination by all pathogens [19]. In both plants, the chilling process with water containing 0.5 to 0.75 ppm of free chlorine was significantly (P < 0.05) associated with an important reduction in *Campylobacter *counts in broilers carcasses. Both, the washing process and the application of chlorinated water during carcass chilling must contribute to these results. Decreases in *Campylobacter *counts associated with chilling operations have also been reported previously, indicating that it is possible to achieve reductions of up to 2 log_10 _CFU of *Campylobacter *on carcasses during processing with chlorinated water [3,20-22]. The results presented here agree with these findings when comparing the median CFU counts per carcass before and after chiller treatment in both plants. Like in the data reported by Stern et al. [22], we found a significant reduction (P < 0.05) not only in the number of *Campylobacter*-positive carcasses but also in the bacterial counts per carcasses, underlining the benefits of an effective washing process of appropriate chlorine concentrations and low temperatures used on a continuous basis in the chiller tanks. The use of chlorinated water during carcass chilling reduced the populations of *Campylobacter*, but this practice, as confirmed in this study, has limited effect in the final magnitude of the *Campylobacter *contamination, because the poultry enter the slaughter processing with a high counts of contamination that the chilling stage is not capable of reducing.

The data presented here confirmed that in our setting a high percentage of commercial chickens are positive for *Campylobacter *at the time of slaughter. As a result, there is a high incidence of *Campylobacter spp*. in retail establishments, this constitute a serious hazard for public health [5,23]. In Chile, Figueroa et al. [24] reported a prevalence of 45% (50/90) of *Campylobacter *contamination in fresh poultry meats. Therefore, reducing the incidence and numbers of *Campylobacter *contamination during the processing of broilers is needed to achieve a safer final product.

## Conclusion

This study has generated data on the high frequency rate of *Campylobacter *contamination in live broiler. This phenomenon derives in high contamination of carcasses and the processing equipment in two Chilean poultry slaughterhouses. According to the data obtained the high rates of cecal carriage at the time of slaughtering is a key factor in the occurrence of *Campylobacter *on both, chicken carcasses and the processing environments. Special attention should be given to the identification of critical control points of potential contamination at the grange level. Also in the processing, such as the plucking and evisceration steps in order to reduce cross contamination with fecal contents during subsequent processes. The data obtained have also shown that the chilling step is a critical control point to reduce carcass contamination but also to reduce the total counts per carcass. With regard to the international importance of the Chilean poultry industry, specially now when chicken exports have experimented strong growth, reaching highly demanding markets such as Mexico, the European Union, China and Japan, among others, information generated by this study may be used as a reference when setting food safety targets, in evaluating individual producers and food safety programs "from the farm to the fork", when HACCP program needs to be scientifically validated and applied more consistently at all stages of poultry production or when designing risk assessment actions. Emphasis should be given to the consumers to cook chicken thoroughly and handle this product carefully as a potential source of *Campylobacter spp*. in order to avoid illness and cross contamination to other food items.

## Methods

### Experimental design

The occurrence of thermotolerant *Campylobacter *contamination in poultry carcasses was evaluated in consecutive samplings in two processing plants (A and B). The samples were randomly collected between January 2006 and January 2007. Each chicken processing plant, located in Santiago Metropolitan Area, was visited on 11 occasions. Plants A and B had processing capacities of 120.000 and 70.000 birds per day, respectively. Both plants have some differences in the processes applied: plant A's chilling process utilizes a dual water tank system with NaClO added followed by air chilling. Plant B's chilling process relies on carcass cooling through water chilling exclusively with NaClO also added. The second difference noted was the timing of the chicken carcasses marinade (salt injection). Plant A marinated the carcasses prior to the chilling process, while plant B marinated them after the chilling process.

### Sample collection

At each sampling, thermotolerant *Campylobacter *contamination was evaluated in four steps during poultry processing: reception (n = 92), after defeathering (n = 124), after evisceration (n = 136) and after chilling (n = 123). Broilers were 42 days old at slaughter and their live weight was 2.5 and 3.5 kg. When carcasses were received, samples were obtained by means of cloacal swabs which were immersed in sterile tubes with 1 ml of 0.1% peptone water. For the remaining 3 stages of bird processing (after defeathering, evisceration and chilling), carcasses were removed from the line at random using a clean pair of latex gloves for each specimen and immediately placed in a sterile plastic bag. On every occasion, broiler carcasses were taken from the same production lot (i.e. birds from the same origin, transported in the same truck and processed in the same conditions). Furthermore, from each plant 20 caecal samples were collected from the evisceration line in sterile plastic bags. To evaluate the possible environment contamination at the processing plant, we analyzed 110 samples directly collected by immersing 500 ml sterile bottles in the scald and in the chill tanks (n = 22 samples), respectively. We also analyzed swab samples from obvious fecal contaminations of the transport crates (n = 22), evisceration machines (n = 22) and defeathering machines (n = 22). In each case, samples were obtained prior to site washing by the plant personnel. All swab samples were placed in sterile tubes containing 1 ml of 0.1% peptone water before inoculation to an appropriate selective culture media.

Following collection, samples were transported at 4°C in refrigerated boxes within 1 h to the Microbiology and Probiotics Laboratory, INTA, University of Chile. The isolation and identification of thermotolerant *Campylobacter *was performed through a validated FSIS method [25]. Bacterial analysis was initiated upon arrival in the laboratory.

To assess the presence of active chlorine in the cooling tanks, free chlorine concentrations were determined "in situ" with a chlorimeter.

### Isolation and identification of thermotolerant *Campylobacter*

#### Whole chicken carcass

To each raw whole chicken carcass 200 ml of 0.1% peptone water were added on arrival laboratory. Carcass rinses were performed by hand shaking for 60 seconds in each of two directions to ensure that the water came into contact with all surfaces. Then, 10 ml of the total volume were centrifuged at 5000 rpm for 5 minutes, and two loops of the centrifugate was streaked on modified Charcoal Cefoperazone Deoxycholate Agar (mCCDA) containing cefoperazone, amphotericin B and rifampicin. The plates were incubated at 42°C for 48 h in gas jars with a microaerobic atmosphere. As an additional enrichment step, 10 ml of each rinse fluid were transferred to 90 ml of Hunt Enrichment Broth (HEB) an incubated at 37°C for 48 h in gas jars with a microaerobic atmosphere (5% O_2_, 10% CO_2 _and 85% N_2_). After incubation, all plates were inspected for suspicious colonies, which were Gram-stained and cell compatible with *Campylobacter *were sub-cultured onto Skirrow agar and incubated for 48 h–5 days at 42°C under microaerobic conditions. All colony types were further identified as *C. jejuni, C. coli*, or *C. lari *using the extended biotyping scheme of Lior [26].

#### Caecal Contents

Thermotolerant *Campylobacter *contamination was evaluated by analyzing approximately 3 cm of the caecal mucosae. The tissue was maintained in a sterile container, inoculated aseptically onto mCCDA plates and incubated under microaerobic conditions at 42°C for 48 h.

#### Processing Plant Environment samples

Swab samples of the transport crates and the defeathering and evisceration machines were examined for *Campylobacter *by direct plating onto mCCDA agar. The plates were then incubated as described above. As for the tank water samples, 10 ml from the scalding and chilling water tanks were transferred to 90 ml HEB enrichment broth and incubated at 37°C for 48 h in gas jars with a microaerobic atmosphere. After enrichment, three loops of the enrichment broth were streaked onto mCCDA and incubated as previously described.

#### Enumeration of thermotolerant *Campylobacter*

Contamination rates with thermotolerant *Campylobacter *after evisceration and chilling were quantified as described by Stern and Pretanik [27]. Briefly, 0.1-ml aliquots of each dilution of the rinse water was plated directly onto duplicate mCCDA agar plates and incubated at 42°C for 48 h under microaerobic atmosphere. All colony types were further confirmed as previously described. Since 0.1 ml of rinse suspension from the total rinse volume of 200 ml was plated, the sensitivity of the method to detect the organism represented an estimated 2,000 CFU per carcass. Counts of CFU at each dilution were averaged, and estimations of *Campylobacter *concentrations per carcass were calculated.

### Statistical analysis

Analysis of differences in the *Campylobacter *culture counts in the different steps during poultry processing was performed using a test of proportion. *Campylobacter *mean counts per carcass following the evisceration and the chilling steps were compared applying the Kruskal-Wallis test. P < 0.05 was considered statistically significant.

## Authors' contributions

GOF conceived the study, participated in its design and approved the final manuscript.

MRT participated in its design, microbiological assays, performed statistical analysis and reviewed the paper. CEL carried out the sample collection, microbiological assays, assisted with the development of methods and wrote first drafts of the manuscript. PCR assisted with the development of methods, microbiological assays and reviewed the paper. MAT performed microbiological assays and statistical analysis.
